# Short-Term Effect of Prebiotics Administration on Stool Characteristics and Serum Cytokines Dynamics in Very Young Children with Acute Diarrhea 

**DOI:** 10.3390/nu2070683

**Published:** 2010-06-30

**Authors:** Nachum Vaisman, Josef Press, Eugene Leibovitz, Güenther Boehm, Vivian Barak

**Affiliations:** 1 Unit of Clinical Nutrition, Tel Aviv Sourasky Medical Center, Sackler Faulty of Medicine, Tel Aviv University, Tel Aviv, 64239, Israel; 2 Pediatric Emergency Room, Soroka University Medical Center, Beer-Sheva, 84101, Israel; Email: press@clalit.org.il; 3 Pediatric Infectious Disease Unit, Soroka University Medical Center, Beer-Sheva, 84111, Israel; Email: eugenel@bgu.ac.il; 4 Danone Research Friedrichsdorf, Germany and Sophia Children’s Hospital, Erasmus University, Rotterdam, 3015GJ, The Netherlands; Email: Guenther.Boehm@danone.com; 5 Immunology Laboratory for Tumor Diagnosis, Oncology Department, Hadassah-Hebrew University Medical Center, Jerusalem, 91120, Israel; Email: barakvivi@hadassah.org.il

**Keywords:** diarrhea, infants, prebiotics, galacto-oligosaccharides, fructo-oligosaccharides, galacturonic oligosaccharides, cytokines

## Abstract

We investigated the effect of a mixture of long-chain fructo-oligosaccharides, galacto-oligosaccharides and acidic oligosaccharides on the number and consistency of stools and on immune system biomarkers in 104 supplemented and non-supplemented subjects (aged 9-24 months) with acute diarrhea. Interleukin-1 (IL-1), IL-1RA, IL-6, IL-8, IL-10, TNF-α and sIL-2R cytokine levels were determined. The significant decrease in number of stools and increase in stool consistency in the supplemented group was of little clinical relevance. The only significant change in pro- and anti-inflammatory cytokines was decreased TNF-α levels in the supplemented group. Prebiotic supplementation during acute diarrhea episodes did not influence the clinical course.

## 1. Introduction

The importance of an intact intestinal microorganism ecosystem is well documented [1]. In breast-fed infants, the intestinal microbiota, characterized by a dominance of bifidobacteria [2], is seen as an important stimulator of the postnatal development of the intestinal barrier function [3] and of the entire immune system [4,5]. It was recently suggested that a modification of the intestinal ecosystem by the intake of dietary components such as probiotics [6] and prebiotics [7] could be beneficial. This ecosystem is vulnerable to pathogenic bacteria and viruses, as well as to drugs, antibiotics and laxatives. During diarrhea, pathogens may either grow and colonize the gastrointestinal (GI) tract and then invade the host tissues or, alternatively, they may secrete toxins which may disrupt the function of the intestinal mucosa, causing nausea, vomiting and diarrhea. Various studies indicated that the administration of probiotics to patients with mild-to-moderate diarrhea can reduce the duration of diarrhea by about one day [8,9]. The clinical relevance of this reduction, however, is still debatable. Prebiotics may also considerably increase the number of specific protective gut microbiota [10], but no data are available on the effect of prebiotics on acute diarrhea in humans. Oli *et al.* [11] showed that adding fructo-oligosaccharides (FOS) to an oral electrolyte solution accelerated the recovery of bacteria, a reaction perceived as beneficial, in an animal experiment. Brunser *et al.* [12] studied the effect of FOS on the intestinal microbiota during treatment with amoxicillin and reported an increase in bifidobacteria in patients receiving prebiotics after seven days of antibiotic treatment compared to a control group. These authors reported that the effect of FOS on the occurrence on antibiotic-related diarrhea episodes was not significant. 

During the last few years, a prebiotic mixture (90% short-chain galacto-oligosaccharides [scGOS) and 10% long-chain FOS (lcFOS)) has been developed as a component in infant formulas. Several studies demonstrated that this mixture modulated the entire microbiota closer to the intestinal flora composition found in breast-fed infants [13]. *In vitro* studies demonstrated that these oligosaccharides inhibit the adhesion of pathogens to the intestinal epithelial surface [14] and enhance a Th1-dependent vaccination response [15], suggesting an anti-infective effect for these oligosaccharides on top of their prebiotic effect. 

In animal experiments [15] as well as in human studies [16,17], the amount of time needed in order to detect significant effect of prebiotics on gut microbiota was at least two weeks, indicating that a preventative approach could be more relevant than a therapeutic approach. The objectives of the current work were to study the possibility of modifying the course of diarrhea in infants and young children by daily supplementation of a prebiotic mixture of neutral (GOS, FOS) and acidic oligosaccharides (AOS) during the first 10 days of diarrhea, and also to investigate the dynamics of interleukin-1 (IL-1), IL-1RA, IL-6, IL-8, IL-10, TNF-α, and sIL-2R serum secretion in response to this supplementation.

## 2. Materials and Methods

### 2.1. Study Population and Study Design

This was a prospective, randomized, double blind placebo-controlled study performed in 2006-2007 at the Pediatric Emergency Room of the Soroka University Medical Center (Beer-Sheva, Israel), where around 60 cases of acute gastroenteritis are seen each month (EL, personal communication). The study was supported by a grant from Danone Research. Parents of infants aged between nine months and two years who had been admitted to the emergency services due to acute diarrhea signed a consent form for their child’s enrollment into this study. Inclusion criteria were no previous history of milk protein allergy or antibiotic treatment, or no chronic GI problems. The study was approved by the hospital ethics committee. 

Stool cultures were performed at admission. Each child was started on a food supplement consisting of three 2-g sachets per day of either maltodextrin (placebo) or a mixture of 80% neutral oligosaccharides (GOS and FOS in a ratio of 9:1) [12], and 20% AOS derived from cleavage of pectin [11]. The supplement was to be taken with three daily meals (a total of 6 g/day). The parents were asked to return to the emergency room with their child on days 2-3, 4-5 and 9-12 after enrollment and to complete a questionnaire on the number of stools and stool consistency (stool consistency scored from 1-5, with 1 = watery, 2 = watery with material, 3 = soft, 4 = formed, regular consistency and 5 = hard). On visits 1 and 2 (days 0 and 2-3), blood was drawn for determination of serum cytokine concentrations. 

### 2.2. Analytical Methods

Serum levels of the tested cytokines (TNF-α, sIL-2R, IL-1, IL-1RA, IL-6, IL-8 and IL-10) were measured by a solid phase ELISA. A high-sensitive immunoassay kit (Quantikine HS R&D system, Minneapolis, MN 55413, USA) was used. This kit employs an amplification system in which the alkaline phosphatase reaction provides a cofactor that activates a redox cycle leading to the formation of a colored product. The secondary enzyme system consists of alcohol dehydrogenase and diaphorase (Amplifier). Fecal microbiota were analyzed from fresh stool samples by conventional plating techniques. The results were expressed as mean ± 1 standard error (S.E.).

### 2.3. Statistical Analysis

The changes in cytokine levels were examined in three ways: (a) difference between day 0 and day 2-3 levels, (b) percentage of the individual change in concentration divided by admission levels, and (c) dichotomously (*i.e.*, an increase *vs*. no change or a decrease). Comparisons between the two groups of patients (non-supplemented *vs*. supplemented) with regard to gender, age, height, number of stools, serum white blood cell and lymphocyte counts, neutrophil percentages and serum cytokine concentrations were performed using unpaired t-tests, Mann-Whitney and Chi-square tests, as appropriate. Comparisons between the two groups with regard to the number of stools and stool consistency over time were by an analysis of variance (ANOVA) with repeated measures. Contrast analysis was used to compare each time point vis-a-vis time of admission. The changes in cytokine concentrations over the first 2-3 days were expressed as a relative percentage change from the admission concentrations (cytokine _day 2-3_- cytokine _admission_/ cytokine _admission_%). Significance was set at ≤0.05. The SPSS (Chicago, IL) for Windows software, Version 13.0 was used for the analysis. A mixed model analysis was performed using the SAS for Windows, version 9.1.

## 3. Results

**Figure 1:**
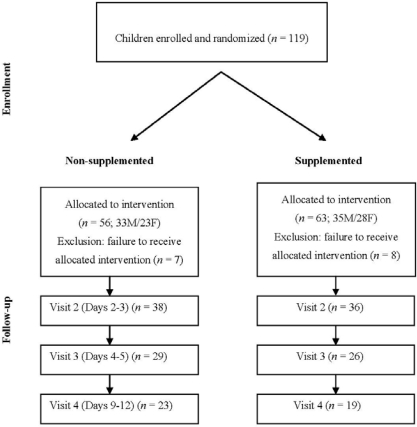
Study flow-sheet: subjects' characteristics and distribution at the follow-up visits.

A total of 119 subjects with acute diarrhea were originally enrolled, but only 104 received the food supplementation due to either referral to hospitalization from the emergency services or withdrawal of consent during the first 24 hours (Figure 1). Out of these 104 patients, 74, 55 and 42 attended visits 2, 3 and 4, respectively (Figure 1). The characteristics of the study participants' evaluated at enrollment are presented in Table 1.

**Table 1:** Patients and stool characteristics upon admission. NS = not significant.

Characteristic	Non-supplemented	Supplemented	P- value
Age, *months*	15.3 ± 9.1	14.5 ± 7.7	NS
Number stools/d	7.34 ± 3.28	6.84 ± 4.41	NS
WBC	11.91 ± 4.30	12.13 ± 4.53	NS
Lymphocytes, *%*	44.48 ± 17.12	39.58 ± 16.01	NS
Granulocytes, *%*	49.54 ± 19.03	53.17 ± 16.92	NS
IL6, *pg/mL*	32.04 ± 58.21	28.76 ± 57.80	NS
IL8, *pg/mL*	108.38 ± 110.54	136.21 ± 204.84	NS
IL10, *pg/mL*	18.86 ± 21.91	16.74 ± 20.40	NS
sIL-2R, *units/mL*	5304.61 ± 1744.67	5936.91 ± 3131.87	NS
TNF-α, *pg/mL*	22.76 ± 8.27	25.55 ± 11.74	NS
IL1-RA, *pg/mL*	12062 ± 20910	14316 ± 18742	NS
IL1-HS, *pg/mL*	3.22 ± 3.04	5.17 ± 5.34	NS

There were no significant group differences for the various studied parameters. Stool cultures were negative in 42 (82.6%) children in the non-supplemented group and in 56 (75.9%) of the children in the prebiotic-supplemented group. Stools were positive for *Campylobacter jejunii* in three non-supplemented children compared to two supplemented children and for *Shigella flexnerii* in one and five, respectively. The stools were not analyzed for the presence of rotavirus or enteroviruses.

**Figure 2:**
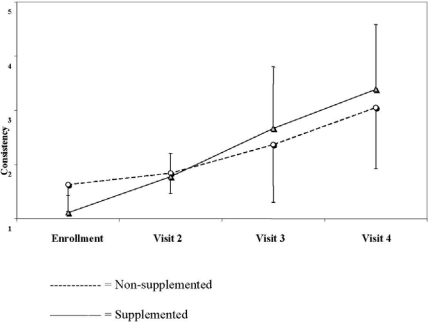
Stool consistency in the two groups over the study period (based on parents' scores from 1 = watery stools to 5 = hard consistency).

The number of stools tended to decrease in both groups during the study period and there were no significant differences between the groups at any of the selected time points (3.1 ± 1.1 stools/day in the non-supplemented group *vs*. 3.3 ± 2.0 stools/day in the supplemented group reported at visit 2, 2.7 ± 1.2 *vs.* 2.4 ± 1.6 reported at visit 3 and 2.3 ± 1.4 *vs.* 2.1 ± 1.7 reported at visit 4, respectively, two-way ANOVA, P = 0.66). Stool consistency increased significantly in both groups during the study period and was significantly higher in the supplemented group (1.8 ± 0.4 *vs.* 1.8 ± 1.1 in the non-supplemented and the supplemented group, respectively, reported at visit 2, 2.4 ± 1.1 *vs.* 2.7 ± 1.1 reported at visit 3 and 3.0 ± 1.1 *vs.* 3.4 ± 1.2 reported at visit 4, respectively P = 0.048) (Figure 2).

**Figure 3:**
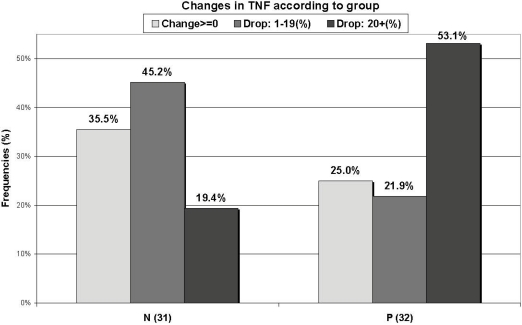
Changes in tumor necrosis factor (TNF-α) levels in each group over the first 48 hours of supplementation. Non-supplemented (N); supplemented (P).

No significant group differences in cytokine serum levels were found at enrollment (Table 1). The cytokine levels changed in all subjects over the first 2-3 days after admission, but the changes were not different between the groups, either in absolute numbers or as a relative change, except for a borderline significant relative change in absolute levels for IL-6 (P = 0.064), indicating a larger decrease in the non-supplemented group. When the subjects with positive bacterial stool cultures were excluded from the analysis, the relative changes in cytokine levels were significantly different for IL-6 (P = 0.043). Since the changes in each group were not uniformly increased, unchanged or decreased, a further dichotomous analysis was done for comparing the percentage of subjects who had a relative increase *versus* no relative change or a relative decrease in serum levels of the different cytokines during the first 2-3 days. The results of the dichotomous analysis indicated that the relative changes in TNF-α concentrations were statistically different between the two groups, with less increase and more decrease in the supplemented group (P = 0.018) (Figure 3), while the change in IL-1 was of borderline significance (P = 0.063), with more increase and less decrease in the values in the supplemented group. Cytokines were also grouped as pro-inflammatory (IL-6, IL-8, TNF-α and IL-1) and anti-inflammatory (IL-1RA, IL-10), but no significant differences were found between these groups. 

## 4. Discussion

Our results indicate that supplementation of prebiotics to the diet of children with acute diarrhea has no significant effect on stool characteristics over the first 10 days of treatment. In fact, data from animals [15] and preterm infants [16,17] indicate that at least 14 days are necessary in order to develop the desired intestinal microbiota. Thus, prebiotics reacting via the development of the intestinal microbiota can preferentially be used for prevention rather than for treatment. 

The next question which was examined in the present study was the value of supplementary prebiotics for influencing the inflammatory process associated with acute diarrhea, as indicated by dynamic changes in cytokine production. Our results do not indicate a significant effect of prebiotics on the cytokine profile during the first 2-3 days of treatment. IL-6 tended to decrease while other cytokines, including some pro-inflammatory ones, such as IL-8 and TNF-α, continued to increase during the first days of diarrhea. No differences were found between the cytokine serum levels of patients treated with a supplementation of prebiotics and those not treated, nor when the different cytokines were sub-grouped as pro- or anti-inflammatory. The change in IL-6 became significantly different when the patients with bacterial gastroenteritis were excluded. Previous studies, published by us [17] and others [18], demonstrated a wide variability in the levels of serum cytokines along the disease course. In some of the patients included in this study, the cytokine levels continued to increase while it had already started to decrease in others to the extent that it was not possible to group the patients. In order to overcome this problem, one of our inclusion criteria was a duration of symptoms of less than 48 hours before the first visit to the emergency room. Nevertheless, a large variability in magnitude and direction of cytokine secretion was found in these patients as well. In an experimental study, Hesse *et al.* suggested that serum cytokines appear in a temporal sequence after endotoxin injection [19]. With this in mind, we further divided each group dichotomously according to the percentage of subjects who had an increase, no change or a decrease in the levels of different cytokines over the first 2-3 days of treatment. The TNF-α level decreased significantly more in the supplemented group (P = 0.018) (Figure 3), but the IL-1 level showed a borderline decrease in the non-supplemented group (P = 0.063). Since TNF-α is an important pro-inflammatory cytokine, this finding may be of clinical importance. 

Different meta-analyses have recently been published on the role of probiotic strains on the course of acute diarrhea [20,21]. Their results showed an overall reduction of one day in the duration of diarrhea in mild-to-moderate episodes but no demonstrable benefit in the more severe cases [22]. The effect was different for different bacterial strains. For example, supplementation with *Lactobacillus paracasei* strain ST11 had no effect on rotavirus diarrhea [23], while *Lactobacillus rhamnousus* did shorten the duration of rotavirus diarrhea but not the diarrhea caused by other pathogens [24]. A recent Cochrane review on the role of probiotics for the prevention of pediatric antibiotic-associated diarrhea (AAD) concluded that probiotics show promise for the prevention of AAD, but the findings were considered too premature for routinely recommending it for prevention [21].

The acute effect of prebiotics in acute diarrhea has not been previously studied. While promising results were found in animal models by using prebiotics to change gut microbiota and prevent AAD, there was no report of successful preventive or therapeutic use of prebiotics in patients with diarrhea and/or inflammatory diseases of the gut [25]. In antibiotic-associated diarrhea, oligo-fructans prevented further episodes of diarrhea in patients with *C. difficile*-associated symptoms treated with metronidazole and vancomycin: this was not found in hospital-based elderly people who were prescribed broad-spectrum antibiotics and received FOS during therapy [26]. Supplementing children aged 1-2 years with a mixture of FOS and inulin after one week of amoxicillin therapy increased fecal bifidobacteria on day 7 of treatment without any apparent change in diarrheal symptoms [11]. Previous publications indicated a possible effect of prebiotics on the immunological response [27,28,29]. Our results did indicate some changes in the cytokine profile: this is intriguing and warrants looking into in order to further explore the inflammatory response associated with acute diarrhea. 

In conclusion, our results showed no significant clinical effect of prebiotic administration during acute diarrhea episodes in children aged 1-2 years. The changes we did observe in cytokine production should be further investigated in order to clarify whether or not prebiotics have any short-term effect in modifying the inflammatory process which accompanies episodes of acute diarrhea. 

## Conflict of interest

NV is a consultant for Danone Research and GB works for Danone.

JP, EL and GB were funded by Danone research (through the principle investigator, NV) for this study.

**No conflict of interest:** JP, EL and VB

## Acknowledgments

The study was supported by Danone research funding. Esther Eshkol is thanked for editorial assistance.
